# Host-probiotic interaction: new insight into the role of the endocannabinoid system by *in vivo* and *ex vivo* approaches

**DOI:** 10.1038/s41598-017-01322-1

**Published:** 2017-04-28

**Authors:** Giorgia Gioacchini, Giacomo Rossi, Oliana Carnevali

**Affiliations:** 1 Dipartimento Scienze della Vita e dell’Ambiente, Università Politecnica delle Marche, Via Brecce Bianche, 60131 Ancona, Italy; 2 Scuola di Bioscienze e Medicina Veterinaria, Università degli Studi di Camerino, Via Fidanza 15, 62024 Matelica, MC Italy; 3 INBB Consorzio Interuniversitario di Biosistemi e Biostrutture, 00136 Roma, Italy

## Abstract

The endocannabinoid system plays an important role in regulating inflammation in several chronic or anomalous gut inflammatory diseases. *In vivo* and *ex vivo* studies showed that 30 days treatment with a probiotic mix activated the endocannabinoid system in zebrafish. These results highlight the potential of this probiotic mixture to regulate immune cell function, by inducing gene expression of toll-like receptors and other immune related molecules. Furthermore, TUNEL assay showed a decrease in the number of apoptotic cells, and this finding was supported by a reduction in pro-apoptotic factors and an increase in anti-apoptotic molecules. The results presented here strengthen the molecular mechanisms activated by probiotic mix controlling immune response and inflammation.

## Introduction

A number of recent findings have shown that the endocannabinoid system (ECS) represents an alternative way to control and regulate the immune system and inflammation^1^. The ECS is a complex endogenous signalling network, which includes cannabinoid receptors (endocannabinoid receptor 1 [Cnr1], endocannabinoid receptor 2 [Cnr2] and capsaicin receptor transient potential vanilloid receptor 1 [Trpv1]), endogenous ligands (anandamide [AEA] and 2-arachidoonoyl-glycerol [2-AG]) that bind and activate the ECS receptors, and enzymes that either synthesize endocannabinoids (abhydrolase domain containing 4 [Abhd4]) or degrade them (fatty acid amide hydrolase [Faah] and monoacylglycerol lipase [Mgll])^2^. The ECS has pro-homoeostatic actions in the brain and the periphery affecting the immune system, autonomic nervous system, endocrine network, reproductive system and gastrointestinal function^1, 3, 4^. Its role in the regulation of the immune system is well established. In particular, it was demonstrated that all immune cells are able to synthetize, degrade or secrete endocannabinoids and possess cannabinoids receptors^5^. It has also been suggested that the ECS controls immune response activity by affecting toll-like receptor (Tlr) induced signalling, which in turn may regulate innate immune cell functioning and subsequent inflammatory events; however, the molecular evidence for this is still weak^6^.

Deficiencies in the ECS are associated with pathological states including irritable bowel syndrome^7^, colorectal carcinoma^8^ and celiac disease^9^. Clinical endocannabinoid deficiency syndromes (CEDS) may be improved by enhancing the ECS. Possible clinical approaches may include drugs or natural products able to increase biosynthesis of ECS ligands or decrease their degradation, or alternatively, modulate the density or function of receptors.

The emerging perception that the gut microbiota plays a central role in regulating many physiological process within the host has supported the importance of the probiotic concept.

Probiotics have been defined as live microorganisms that, when administered in proper amounts, improve host health^10^. The role of the intestinal microbiota in the modulation of the ECS and intestinal pain has been demonstrated in different experimental models^11, 12^. The probiotic formulation selected for this study was VSL#3 containing 450 billion of a mixture of *Lactobacilli*, *Bifidobacteria*, and *Streptococcus thermophilus* (commercialized in EU as Vivomixx® and in USA as Visbiome®). This probiotic mixture was selected for this study because of its recognized clinical potential in the treatment of several gastrointestinal inflammatory diseases^13–15^. Although the mechanism of action of VSL#3 is not fully understood several studies performed on animal models have shown that VSL#3 modulates the host immune response. In particular VSL#3 is a potent inducer of IL-10 in intestinal and blood dendritic cells and inhibits the generation of pro-inflammatory T helper cells^16^. It appears the protective effects of VSL#3 may be mediated by the DNA isolated from bacterial components of VSL#3 rather than by their metabolites or ability to colonise the colon. It was demonstrated that the anti-inflammatory effect of VSL#3 was dependent on non-methylated bacterial DNA (CpG) signalling acting via the toll-like receptor (TLR)-9^17^. The aim of the study was to provide, by *in vivo* and *ex vivo* experiments, new insights into the molecular mechanisms through which VSL#3 probiotics can be recognized as non-pathogen microbes and can activate the immune response without causing inflammation. The zebrafish, *Danio rerio*, has gained considerable attention as an experimental model in biomedical research^18^. The availability of germ-free species, the resemblance of its physiology to that of mammals, the high similarity of immune system molecules to those in other vertebrates including mammals^19–21^, together with the high throughput capability in genetic and chemical screens, give zebrafish research a significant impact in promoting the development of personalized and precision medicine. Zebrafish colonization is characterized by a condition very similar to that of human. To investigate the effect of microbial colonization on the zebrafish host, Rawls and colleagues^22^ reared germfree zebrafish and investigate gene expression profiles. They showed that colonization altered the expression of 212 genes. Of these 212 genes, 59 responses were conserved between human, mice and zebrafish. These conserved genes were mainly involved in epithelial proliferation, promotion of nutrient metabolism, and innate immune responses. This indicates that the response towards microbes is in part highly conserved in all these species, especially the pathways here studied.

## Results

### Effects of VSL#3 on intestinal epithelium integrity

**Figure 1:**
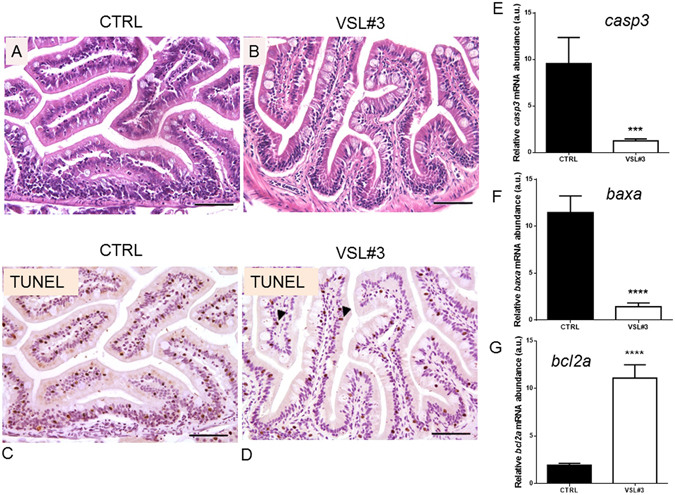
(**A**) Paraphysiological morphology of intestinal wall in untreated control zebrafish (bar = 200 µm). (**B**) Morphology of intestine of VSL#3-treated zebrafish reveals good integrity of the gut mucosa, without any inflammatory infiltrate or reactive status to probiotics (bar = 200 µm). (**C**) In section of gut of untreated fish, a high apoptotic rate evidenced by TUNEL is evident among epithelial and mesenchymal cells, with nuclear intense black stain (bar = 200 µm). (**D**) TUNEL analysis of section of VSL#3-treated intestine revealed that some enterocytes and mesenchymal cells are going into apoptosis, showing their nuclei intensively black stained by the chromogen (arrow heads, bar = 200 µm). Intestinal (**E**) *casp3*, (**F**) *baxa* and (**G**) *bcl2a* mRNA levels by qPCR normalized against *act1b* and *rplp*, in control and VSL#3-treated fish. Values indicate mean ± SD. ***p < 0.001 ****p < 0.0001.

Healthy fish exposed to VSL#3 for 4 weeks showed similar behaviour and survival to controls. Morphological study of intestines of VSL#3-treated zebrafish revealed substantial integrity of gut mucosa comparable to that of controls, without any inflammatory infiltrate or reactive status to probiotics (Fig. 1A,B).

The histological analysis showed an intact epithelial barrier with a columnar uniform epithelial and regular apical brush border without signs of damage in the intestines exposed to probiotic. In addition, after VSL#3 treatment significant differences were seen in enterocyte length, villus length and crypt depth (Table 1S).

Moreover, TUNEL assay showed a high apoptotic rate among epithelial and mesenchymal cells in gut from control fish with respect to treated ones (Fig. 1C,D). The reduction in the apoptotic process in intestinal epithelium after probiotic administration was also confirmed at molecular level by the lower expression of pro-apoptotic signals such as *casp3* and *baxa*, concomitantly with an increase in anti-apoptotic signals such as *bcl2a* (Fig. 1E–G).

### Effects of VSL#3 on the endocannabinoid system: an *in vivo* approach

**Figure 2:**
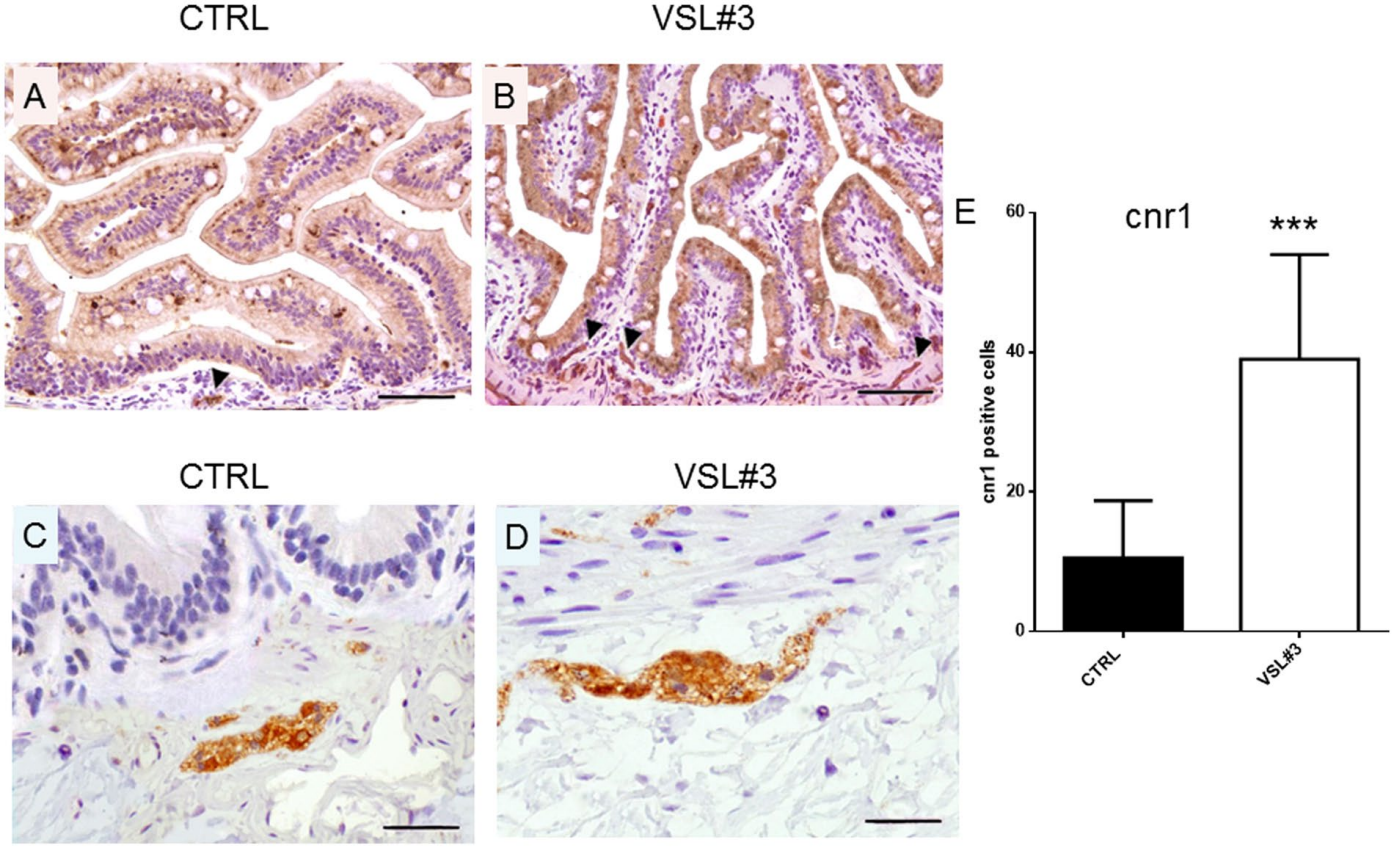
(**A**,**C**) Section from untreated fish, stained for cnr1 receptor, shows a strong stain restricted to parietal ganglia only (arrow head), with low levels of expression in enterocytes or other cell types (A-bar = 200 µm; C- bar = 50 µm). (**B**) Strong expression of cnr1 is observed in epithelial cells, in some mesenchymal cells, and in ganglia (arrow heads) of the muscular layer of intestine wall of VSL#3-treated fish (bar = 200 µm). (**D**) Note the strong presence of the receptor in a ganglion, as demonstrated by high power magnification of a particular area in the same section (bar = 50 µm). (**E**) Intestinal cnr1 protein levels by IHC in control and VSL#3-treated fish. Values indicate mean ± SD. ***p < 0.001.

**Figure 3:**
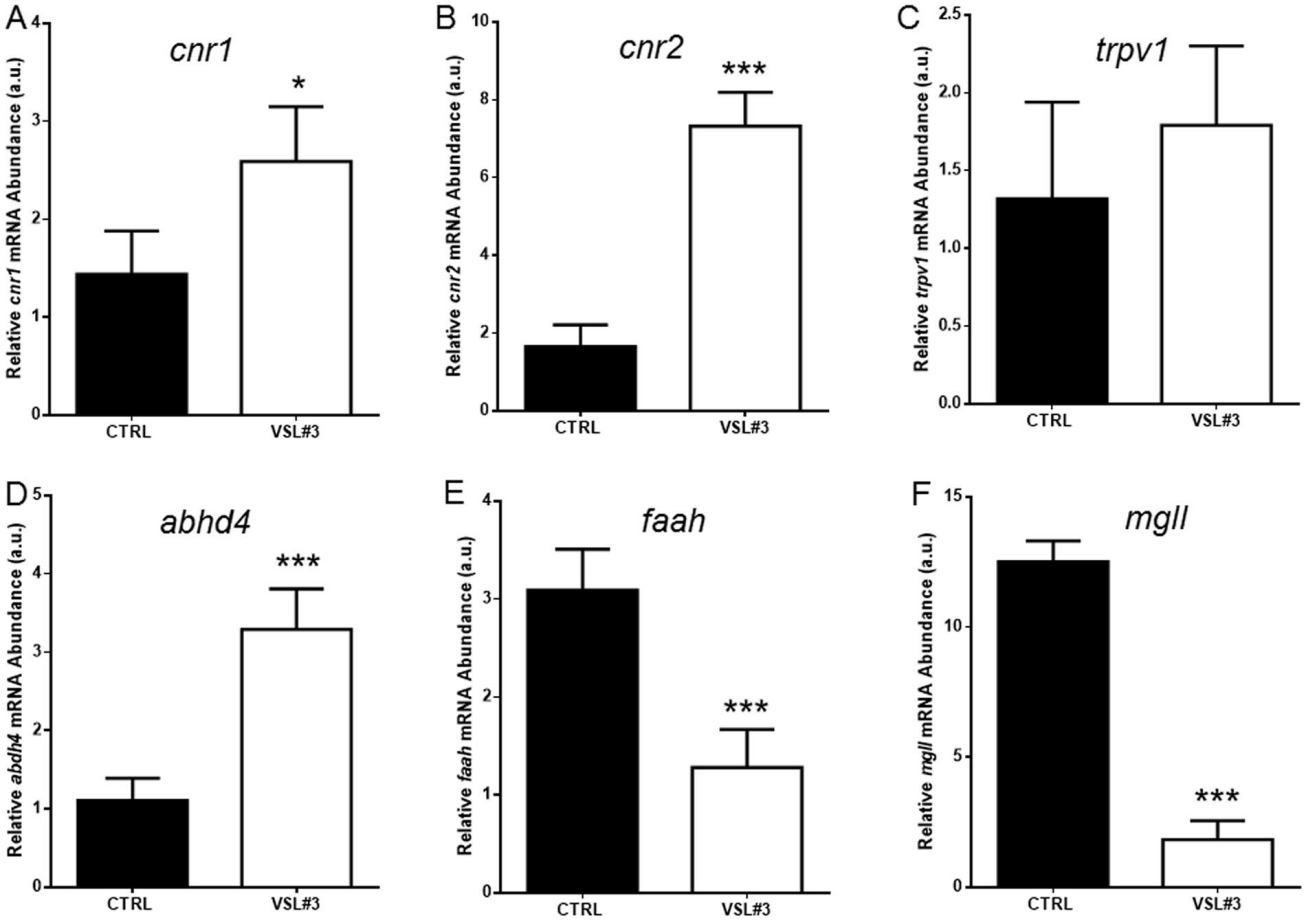
Intestinal (**A**) *cnr1*, (**B**) *cnr2*, (**C**) *trpv1*, (**D**) *abhd4*, (**E**) *faah* and (**F**) *mgll* mRNA levels by qPCR normalized against *act1b* and *rplp*, in control (CTRL) and VSL#3-treated (VSL#3) fish. Values indicate mean ± SD. *p < 0.05; ***p < 0.001.

Figures 2 and 3 show modulation of the ECS after VSL#3 administration. IHC analysis of gut sections from treated animals highlighted strong presence of Cnr1 in epithelial cells, in some mesenchymal cells, and in the ganglia of the muscular layer of the intestine wall. In contrast, gut sections from control animals showed a strong stain only in parietal ganglia, with markedly low levels of expression in enterocytes or other cell types (Fig. 2A–E). At molecular level, the involvement of VSL#3 administration on endocannabinoids synthesis and degradation was shown firstly in the gut. A significant increase of *abhd4* gene expression was observed, coding for a key enzyme involved in AEA synthesis. Concomitantly, gene expression of *faah* and *mgll*, which code for the enzymes involved in degradation of AEA and 2-AG respectively, was significantly decreased after VSL#3 treatment. Moreover, molecular changes to endocannabinoid receptors *cnr1* and *cnr2* in zebrafish gut as consequence of VSL#3 administration were also observed while, the *trpv1* gene was not affected (Fig. 3A–F).

### Effects of VSL#3 on toll-like receptor activation

In zebrafish intestine, 30 days of probiotic administration induced a significant (p < 0.001) increase in gene expression levels of key molecules that can recognize microbes in the intestine such as *tlr1*, *tlr2* and *tlr9*, with respect to control animals (Table 1A). The increase in gene expression was consistent with the increase at protein level found by immunohistochemistry with the exception of Tlr2, which showed no significant changes (Table 1B).

**Table 1:** (A) Intestinal mRNA levels tested by qPCR and (B) protein levels by IHC of immune system and inflammatory related molecules, in control (CTRL) and VSL#3-treated (VSL#3) 129 fish.

Gene name	Pathway involved	Control	VSL#3
*Il1b*	Inflammatory cascade	1.29 ± 0.33	14.51 ± 2.69***
*tnfa*	Inflammatory cascade	1.04 ± 0.21	2.59 ± 0.44***
*myd88*	Production of pro-inflammatory cytokines	1.24 ± 0.36	3.15 ± 0.35***
*Il10*	Anti-inflammatory action	1.54 ± 0.84	13.58 ± 2.89****
*casp1*	Stimulation of cell survival responses, control intracellular bacterial growth and modulation of inflammatory cytokine production	2.14 ± 0.84	8.75 ± 1.89****
*nos2a*	Protective role in intestinal inflammation	1.09 ± 0.21	3.73 ± 0.30***
*tgfb1a*	Anti-inflammatory action	1.29 ± 0.12	3.02 ± 0.37****
*nfkb*	Production of pro-inflammatory cytokines	1.48 ± 0.36	2.98 ± 0.21***
*tlr1*	Recognition of cell-surface bacterial peptidoglycans	2.08 ± 1.02	36.75 ± 4.17***
*tlr2*	Recognition of cell-surface bacterial lipoproteins	1.30 ± 0.35	19.59 ± 3.35***
*tlr3*	Nucleic acid-sensing and recognition nucleotides from both viruses and bacteria	1.12 ± 0.36	13.22 ± 3.74*
*tlr9*	Nucleic acid-sensing and recognize nucleotides from both viruses and bacteria	1.42 ± 0.51	17.07 ± 2.43*
Protein name		Control	VSL#3
Il1b	Inflammatory cascade	7.46 ± 3.23	24.26 ± 6.30***
Tnfa	Inflammatory cascade	38.23 ± 13.24	94.26 ± 16.37***
Myd88	Production of pro-inflammatory cytokines	9.06 ± 4.23	23.33 ± 6.30**
Il10	Anti-inflammatory action	12.58 ± 4.33	32.58 ± 3.69****
Casp1	Stimulation of cell survival responses, control intracellular bacterial growth and modulation of inflammatory cytokine production	9.85 ± 3.84	51.68 ± 15.63***
Nos2a	Protective role in intestinal inflammation	2.61 ± 2.41	31.55 ± 10.51****
Tgfb1a	Anti-inflammatory action	2.06 ± 1.27	43.84 ± 16.74****
Nfkb	Production of pro-inflammatory cytokines	38.71 ± 15.43	87.72 ± 16.47***
Tlr1	Recognition of cell-surface bacterial peptidoglycans	5.35 ± 3.55	24.55 ± 5.57****
Tlr2	Recognition of cell-surface bacterial lipoproteins	9.61 ± 4.43	15.35 ± 8.94
Tlr3	Nucleic acid-sensing and recognition nucleotides from both viruses and bacteria	1.45 ± 1.01	21.66 ± 9.55****
Tlr9	Nucleic acid-sensing and recognize nucleotides from both viruses and bacteria	20.43 ± 9.92	39.92 ± 12.44****

Values indicate mean ± SD. *p < 0.5; **p < 0.01; ***p < 0.001; ****p < 0.0001.

**Figure 4:**
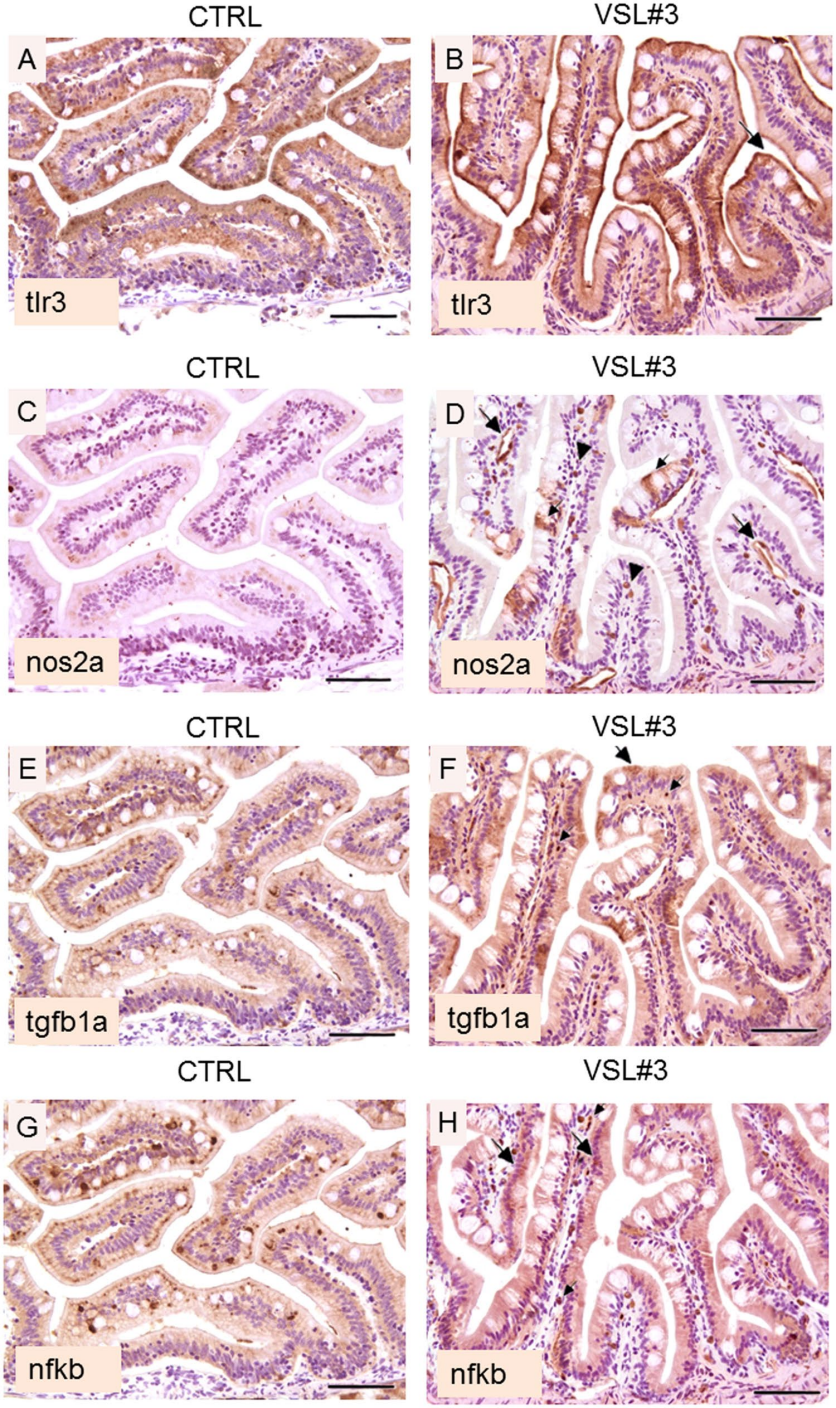
(**A**) Intestine section from untreated fish; note the evident reduction in intensity of tlr3 staining with respect to VSL#3-treated fish, characterized by a weak and irregular enterocyte expression, without any staining of cell free pole membranes (bar = 200 µm). (**B**) Intestine section from treated fish; note the intense and diffuse presence of tlr3 in the mucosal epithelium. The stain is almost exclusively restricted along the superficial margin of enterocytes (arrow heads), and shows a continuous and constant pattern (bar = 200 µm). (**C**) In section from untreated fish, note the substantial negativity for the presence of Nos2a, both in the epithelium and in endothelial cells (bar = 200 µm). (**D**) In intestine section from treated fish, an evident presence of Nos2a is observed, especially in endothelial cells (large arrows) or in scattered mononuclear cells (presumably macrophages) interspersed throughout the mucosal chorion (arrow heads). Note the molecule is also localized in some epithelial cells (small arrow); (bar = 200 µm). (**E**) Untreated zebrafish show low level of immunoreactivity for Tgfb1a both in the epithelium and in mononuclear cells (bar = 200 µm). (**F**) In VSL#3-treated fish, intestinal presence of Tgfb1a was strong and diffuse throughout the epithelium (large arrows) and in some mononuclear cells (small arrows); (bar = 200 µm). (**G**) In untreated fish the localization of the p65 subunit of the Nfkb heterodimer is restricted to scattered epithelial cells (bar = 200 µm). (**H**) Similarly to Tgfb1a localization, the p65 subunit of the Nfkb heterodimer is present in a large proportion of enterocytes (large arrows). Also note the presence of this marker in many mononuclear cells (small arrows), infiltrating the lamina propria of the intestinal mucosa of VSL#3-treated zebrafish (bar = 200 µm).

IHC analysis showed intense and diffuse presence of Tlr3 in the mucosal epithelium of treated animals. In sections from these animals it is evident that the stain was present in a continuous and constant pattern and almost exclusively localized along the superficial margin of enterocytes. In contrast, sections from control animals were characterized by weak and irregular Tlr3 presence in the enterocyte, without any staining of cell free pole membranes (Fig. 4A,B).

### Effects of VSL#3 on immune response activation

Figure 4 and Table 1A,B illustrate modulation of the immune response after VSL#3 administration. In particular, gut from treated animals showed an increase in both gene and protein levels of molecules implicated in the innate immune response such as Il1b, Tnfa and Myd88 (Table 1A,B). In addition, both mRNA of *il10* and *casp1*, and their protein levels, were significantly (p < 0.001) higher in VSL#3-treated fish than controls (Table 1A,B). Using IHC analysis on gut from treated animals, Nos2a protein was localized specifically in endothelial cells or in macrophages, and to a lesser extent in epithelial cells. In control gut there was substantial negativity for the presence of Nos2a, both in the epithelium and in endothelial cells. (Fig. 4C,D); conversely, an increase of both protein and mRNA level for Nos2a was seen in VSL#3-treated fish (Table 1A,B). Concomitantly, the increase at both gene and protein level of signals involved in antigenic “tolerance” such as Tgfb1a and Nfkb was registered (Table 1A,B). In VSL#3-treated fish, IHC analysis showed higher Tgfb1a protein signal that appeared diffusely localized throughout the epithelium and in mononuclear cells with respect to control ones (Fig. 4E,F). Finally, the p65 subunit of the Nfkb heterodimer was found to be localized in a large proportion of enterocytes and in many mononuclear cells infiltrating the *lamina propria* of intestinal mucosa of VSL#3-treated fish, while in control gut it was restricted to scattered epithelial cells (Fig. 4G,H).

### Effects of VSL#3 on the endocannabinoid system: an *ex vivo* approach

**Figure 5:**
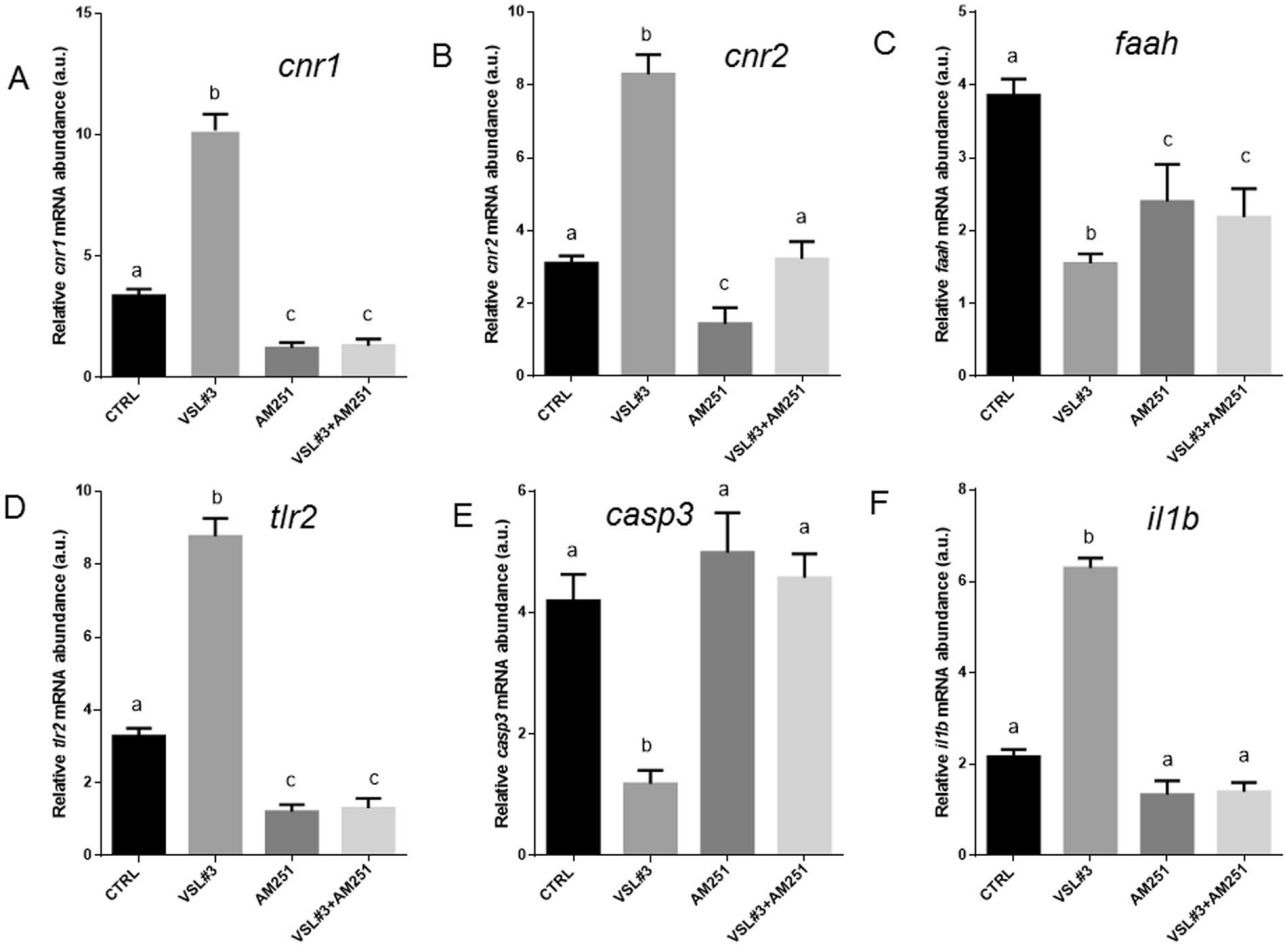
Intestinal mRNA levels evaluated by qPCR of (**A**) *cnr1*, (**B**) *cnr2*, (**C**) *faah*, (**D**) *tlr2*, (**E**) *casp3* and (**F**) *il1b* normalized against *act1b* and *rplp*, in different experimental groups from *ex vivo* experimentation. Values indicate mean ± SD. Different letters denote significant differences among groups.

**Figure 6:**
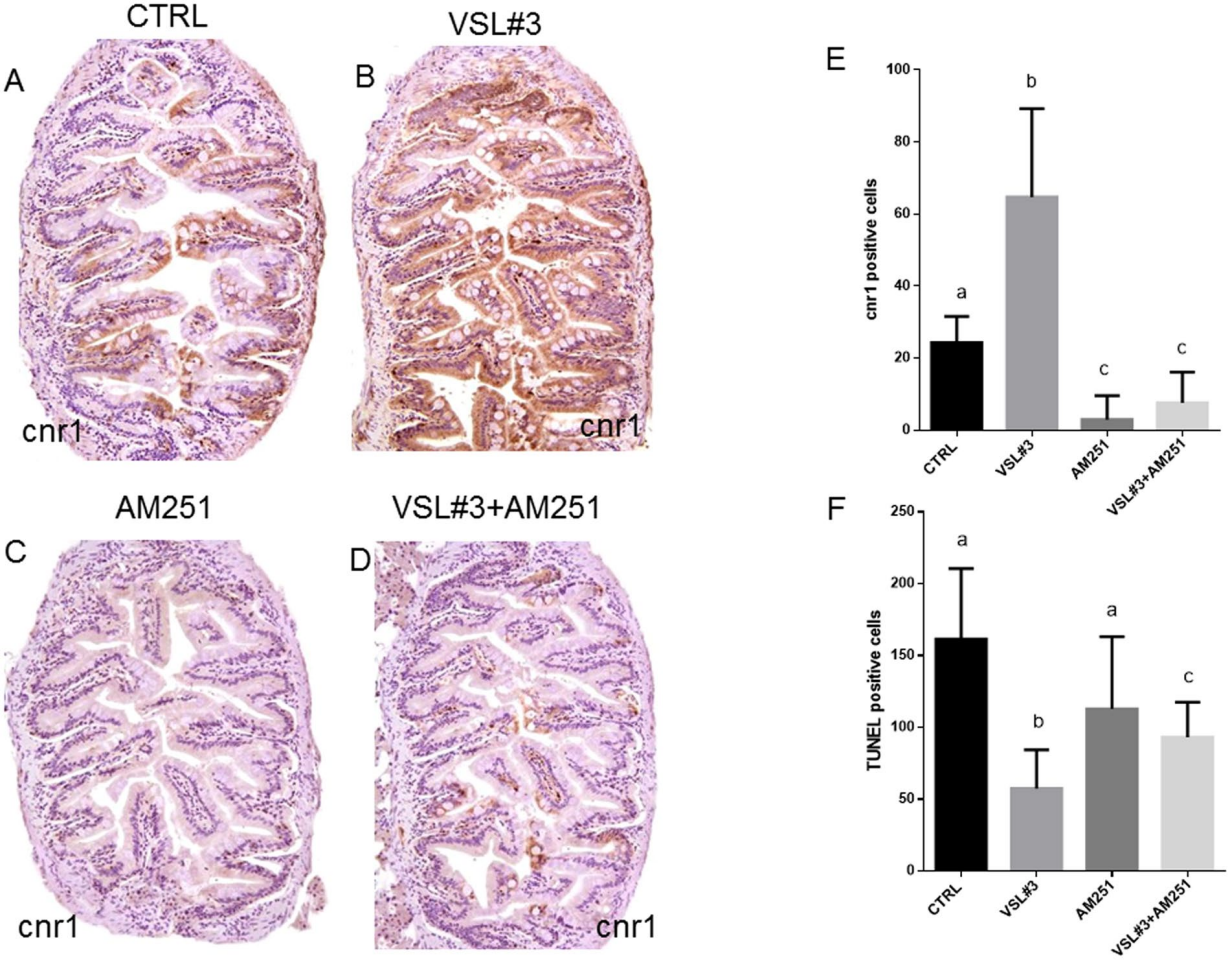
Intestinal distribution of cnr1 by IHC in different experimental groups from *ex vivo* experimentation: (**A**) Control; (**B**) VSL#3; (**C**) AM251; (**D**) VSL#3 + AM251. (**E**) Cnr1 protein levels by IHC and (**F**) TUNEL positive cell count in the same *ex vivo* experimental groups. Values indicate mean ± SD. Different letters denote significant differences among groups.

Figures 5 and 6 illustrate modulation of ECS, immune response and inflammation after *ex vivo* exposure of zebrafish intestine. Eight hours of *in vitro* VSL#3 treatment on *ex vivo* intestine samples significantly increases *cnr1*, *cnr2*, *faah*, *tlr2*, *il1b* and *casp3* gene expression, confirming the stimulatory role of the probiotic. In contrast, 8 hours of exposure to AM251, a Cnr1 antagonist, induced in these samples a significant reduction of *cnr1*, *cnr2*, and *il1b* expression, without affecting *faah* and *casp3* gene expression or the apoptotic rate evidenced by TUNEL. Finally, the concomitant exposure of intestine to VSL#3 and AM251 in the *ex vivo* assay showed a significant decrease in *cnr1*, *tlr2* and *il1b* gene expression and in apoptotic rate, without affecting *cnr2*, *faah* and *casp3* gene expression (Figs 5 and 6).

## Discussion

Chronic or anomalous gut inflammation is the primary cause of many gut inflammatory diseases, including ulcerative colitis and Crohn’s disease, and it has been clearly shown that the endocannabinoid system plays an important role in modulating this inflammation^9, 23–25^. In particular, Di Marzo and Izzo^26^ demonstrated that *cnr1* and *cnr2* activation may limit inflammation on well-established models of IBD in rodents. Data from several trials (reviewed by Chapman *et al*.^13^) suggested that VSL#3 has clinical potential in the treatment of many gut inflammatory diseases, but the mechanisms by which it exerts its beneficial role are not fully elucidated.

Until now no publications are reported in literature, that indicate studies regarding endocannabinoid system organization in gastrointestinal tract and its modulation by the microbiome in zebrafish. Meanwhile, given that several gut functions and immune genes are conserved between zebrafish and mammals, the zebrafish is an interesting model organism to investigate fundamental processes underlying intestinal inflammation and injury^27^. In this context, the similarities of this model with mammal and rodent strongly suggest to consider relevant our results regarding distribution and role of endocannabinoid receptors in ZF. These results are relatable to that which would be the endocannabinoid expression in humans or mice GI tract. The results obtained here in zebrafish intestine are in agreement with the previous reports from Rousseaux and collaborators^11^ done both *in vitro* in human HT-29 epithelial cells and both *in vivo* in Balb/c mice and Sprague Dawley rats, showed the ability of *Lactobacillus acidophilus* to induce intestinal cannabinoid receptors, and from Distrutti and co-workers^28^ who reported increased *cnr1* gene expression in mouse colon after VSL#3 administration.

In the present study, we offer evidence that in control intestine, cnr1 presence was only detectable in ganglia, while in VSL#3-treated gut, Cnr1 was also present in epithelial cells and in some mesenchymal cells. Interestingly, as suggested by Sibaev *et al*.^29^ it should be noted that in our experiments, spindle-shaped cnr1 immunopositive cells, encountered within the outer muscle layer, were systematically ckit/GFAP/S100 immunonegative (data not shown), demonstrating that interstitial cajal cells (ICC) are not involved in this VSL#3-induced *cnr1* overexpression.

The anti-inflammatory capability of VSL#3 was indicated by the positive modulation of *cnr2* concomitantly with the constant level of *trpv1* genes on zebrafish intestine. In fact, the importance of Cnr2 receptor activation in the immune-modulatory effects of endocannabinoids has been well recognized, and its anti-inflammatory role is evident in many pathological conditions such as inflammatory pain and gastrointestinal inflammatory disorders^30^. In addition, it has been shown that Trpv1 binding AEA induces inflammation in the gut^31^.

In the present study, it was demonstrated that in zebrafish, probiotics included in the VSL#3 formulation communicate with the host by activating Tlrs as demonstrated in others species^17^. Chu and Mazmanian^32^ noted in a review that Tlrs, being pattern-recognition receptors (PRR), may have evolved to mediate the bi-directional cross talk between “good” bacteria and their hosts. In particular, in zebrafish the PRR system is highly similar to that in other vertebrates and *tlr* orthologs have been identified and well characterized in this fish species^33, 34^. Key features of the fish Tlrs and the factors involved in their signalling cascade have high structural similarity to the mammalian Tlr system. The ligand specificity of several Tlr receptors is highly conserved in all vertebrates^35^. Most vertebrate genomes are recognized to have at least one gene representing each of the seven major *tlr1*, *tlr2*, *tlr3*, *tlr4*, *tlr5*, *tlr7* and *tlr11* families^36^ but the most conserved among zebrafish and human and other mammals models are *tlr1*, *2 3* and *9* ^34^. In particular, after microbial identification, all Tlrs with the exception of Tlr3 trigger the activation of intracellular pathways, which is myeloid differentiation factor 88 (Myd88)-dependent^37^. In this case, Myd88, through the activation of nuclear factor Nfkb, induces production of pro-inflammatory cytokines including Il1b and Tnfa^37^. The interaction of a probiotic, such as *E*. *coli* Nissle with Tlr2 and Tlr4 was previously found in mammals^38^, but here for the first time we have established the ability of VSL#3 to communicate with the host by activating several tlrs in zebrafish. In particular, VSL#3 administration activated Tlr1 and 2, respectively involved in the recognition of cell-surface bacterial peptidoglycans and lipoproteins both in mammal and fish^39^. Moreover, Tlr3 and 9, which are nucleic acid-sensing and recognize nucleotides from both viruses and bacteria^17, 40^, were activated after VSL#3 administration. The activation of these receptors by VSL#3, in particular Tlr2 and Tlr9, has been related to immune system activation without triggering a negative inflammatory response. Tlr2 is widely described as a PRR, mediating host protective immune responses^41, 42^. Interestingly, not all *Lactobacilli* strains can activate Tlr2, suggesting that strain-specific immunoregulatory effects may be partially mediated via Tlr2 activation^43, 44^.

Results obtained with our zebrafish model, when treated with VSL#3, show on the one hand an over-stimulation of *tlr2* and *tlr9* and respective proteins, and on the other hand increased expression of some inflammatory cytokines, but without histological signs of mucosal inflammation and damage. These results are in line with previous observations in macrophages on the ability of some probiotics, such as *L*. *rhamnosus GG*, to moderately activate the production of inflammatory cytokines and the transcription factors involved in humans^45–48^, but at the same time to prevent a detrimental intestinal inflammatory response. Bacteria contained in the VSL#3 mixture (*Streptococcus thermophilus* DSM 24731, *Bifidobacterium longum* DSM 24736, *Bifidobacterium breve* DSM 24732, *Bifidobacterium infantis* DSM 24737, *Lactobacillus acidophilus* DSM 24735, *Lactobacillus plantarum* DSM 24730, *Lactobacillus paracasei* DSM 24733, *Lactobacillus delbrueckii subsp*. *bulgaricus* DSM 24734), similarly to LC705 and GG, are able to upregulate genes and protein levels for inflammatory cytokines Il1b and Tnfa in zebrafish, as recently reported in mice^49, 50^. Moreover, anti-inflammatory genes such as *il10* and *tgfb1a* and genes for transcription factors such as *nfkb*, were affected by probiotic administration^51^. The anti-inflammatory effect exerted by VSL#3 highlighted in our study was supported by the maintenance of intestinal epithelial integrity, shown by histological analysis. In addition, the reduced apoptosis rate shown by TUNEL assay, as well as the reduced level of pro-apoptotic signals such as *casp3* and *baxa*, concomitantly with the increase in anti-apoptotic signals such as *bcl2a* ^52–54^ support the anti-inflammatory role of VSL#3. Although single epithelial cell apoptosis and elimination without loss of barrier permeability are normal physiological events in the gastrointestinal tract^55^, an increased epithelial apoptotic ratio might be cause of relevant leaks in the epithelial barrier. Uncontrolled cell cycle or apoptosis are often the main mechanisms in pathogenesis of cancers, autoimmune diseases or chronic inflammation^56–58^. Our results are in agreement with some previously obtained in a murine model after VSL#3 treatment showed a decreased apoptosis in intestinal epithelial cells^59^.

In our results, the induction by VSL#3 administration of *il1 and tnfα* associated with *casp1* expression resulted in an anti-apoptotic final outcome. These results are in accordance with the recent observation that *casp1* activation fails to trigger pyroptosis in all cell types, and somewhat surprisingly, epithelial cells use *casp1* activation to prevent cell death^60^. The function of *casp1* is analogous to the activities of other apoptotic caspases (Casp3 and 8) in modulating the fate of certain cell types^61^. Low levels of *casp1*, as probably occurred in our case, stimulate cell survival responses, control intracellular bacterial growth and mediate inflammatory cytokine production. When *casp1* activation reaches a critical threshold level, cells undergo pyroptosis and release inflammatory intracellular contents. To our knowledge, our study is the first to conduct an *ex vivo* study of zebrafish intestine. We compared histological integrity and apoptotic signals in control intestine before and after 8 hours of *in vitro* culture and no significant differences were found, clearly indicating that such culture methods are a valid and reliable tool to study the direct effects of bacteria on intestine.

The results achieved by the *ex vivo* studies clearly showed that the immune stimulant and anti-inflammatory role of VSL#3 is mediated by the activation of the endocannabinoid system. After 8 hours of *in vitro* VSL#3 exposure, a clear induction of *cnr1*, *cnr2*, *tlr2* and *il1b* genes was found, concomitantly with a reduction in *casp3* gene expression and TUNEL positive cells, confirming the *in vivo* study results. At the same time, the lower *cnr1* levels induced by AM251 exposure in cultured intestine were related to a decrease in immunerelated gene expression and an increase in apoptotic signal (*casp3* and TUNEL-positive cells). The simultaneous addition of VSL#3 and AM251 to the culture medium did not re-establish the levels of signals found in the intestine exposed to VSL#3, strengthening the concept that it acts through the activation of endocannabinoid system.

Concluding, the endocannabinoid system is recognized as an anti-inflammatory agent, with identified protective roles in many inflammatory diseases. In the present study, we report *in vivo* and *ex vivo* results that provide a novel molecular network through which probiotics such as VSL#3 activate the endocannabinoid system and induce Tlr signalling. These results further highlight the potential of this probiotic mixture to regulate immune cell functioning, decreasing detrimental effects and consequent inflammatory events in zebrafish. Considering the availability of a zebrafish model of IBD and related diseases^62^, the results discussed here could be the basis for further studies on IBD induced zebrafish.

## Methods

### *In vivo* fish maintenance and VSL#3 treatment

Adult male zebrafish specimens purchased from a local supplier (Acquario di Bologna, Bologna, Italy) were acclimated to laboratory conditions and their health state was monitored for four weeks prior to the beginning of the experiments.

Fish were divided into a control group (CTRL), which was fed with commercial food, and a probiotic-treated group (VSL#3), that received a commercial diet containing the lyophilized probiotic at a final concentration of 10^9^ CFU/g for 30 days. The probiotic formulation utilized was VSL#3 (produced before January 31, 2016; VSL Pharmaceuticals, Inc., Gaithersburg, MD, USA). Probiotics contained 450 × 10^9^ live bacteria (*Streptococcus thermophilus* DSM 24731, *Bifidobacterium longum* DSM 24736, *Bifidobacterium breve* DSM 24732, *Bifidobacterium infantis* DSM 24737, *Lactobacillus acidophilus* DSM 24735, *Lactobacillus plantarum* DSM 24730, *Lactobacillus paracasei* DSM 24733, *Lactobacillus delbrueckii subsp*. *bulgaricus* DSM 24734).

The probiotic strain was mixed into the diet prior to feeding. All fish were served with a quantity of food ranging from 1.5% to 2% of their bodyweight per day. The experiment was conducted in triplicate and for each replicate tank, the final housing density was 20 males.

All the procedures involving animals were conducted in accordance with the Italian law on animal experimentation and were approved by the Ethics Committee of Università Politecnica delle Marche (Prot #63/INT/CESA12-16). All efforts were made to minimize suffering.

After 30 days of treatment, ten males from each experimental group were sacrificed by a lethal overdose of anesthesia (500 mg/L MS-222 [3-aminobenzoic acid ethyl ester] buffered to pH 7.4; Sigma).

Five intestines from each experimental group were removed and fixed in PFA fixative. The remaining intestines were sampled for histology, immunohistochemistry and q-PCR assay.

### *Ex vivo* tissue culture

Twenty-four control fish were sacrificed after a 12-hr fast by a lethal overdose of anesthesia (as above). Intestines were excised and placed in a tissue culture dish of 35 × 10 mm (Falcon). The medium employed in these cultures consisted of 9 parts Dulbecco’s modified medium (DMEM)/Ham’s F12 (Sigma) and 1 part fetal calf serum (Sigma). In addition, 10,000 U of crystalline penicillin G and 0.01 g of streptomycin sulfate (Sigma) were added to each 100 ml of medium. The intestines were cultured in the presence of antibiotics for 4 hours and then placed in a new tissue culture dish in the same medium without the antibiotics. For experimental purposes, incubations were carried out separately in Dulbecco’s Modified Eagle Medium (DMEM) (Thermo Fisher Scientific) (CTRL), DMEM + VSL#3 (final concentration 10^9^ CFU/ml) (VSL#3), DMEM + AM251 (Cayman Chemical) (10 nM) (AM251), and DMEM + VSL#3 + AM251, (VSL#3 + AM251). AM251 concentration was chosen on the basis of previous studies^63, 64^.

The dishes were covered, placed on a steel rack set in a McIntosh jar (Scientific Products, Inc., Los Angeles, CA) and maintained at 37 °C. The jar was gassed with 95% 0_2_ and 5% C0_2_ for 20 min and then sealed. Intestines from each experimental group were sampled for histology, immunohistochemistry and q-PCR assay after 8 hours of culture.

A 5 mm-long piece from the anterior part of each *ex-vivo* cultured gut was used also for morphological evaluations, after dissection and a 10% buffered formalin fixing procedure and immunohistochemistry analysis.

### Histology

PFA-fixed samples were processed for routine histology to obtain a transversal section, which was stained with hematoxylin and eosin (H&E). Sections were evaluated under a light microscope (Carl Zeiss, Jena, Germany) for any degenerative and/or inflammatory changes.

Villus length, crypt depth, villus length/crypt depth ratio and enterocyte length were determined from three fish per group; 50 images were taken for each intestine, for a total of 150 images per group, and 12 enterocytes were measured per image. Enterocyte lengths were measured starting from their basal membrane connected with the lamina propria up to the free, luminal pole of the cell. All measurements were taken from micrographs using the Software Image J. In all cases, measurements were made using sections in which villi, cryptae, and enterocytes were cut along their entire length; only crypts or villi that were sagittally sectioned along their entire length were scored. For enterocytes, the cell was considered complete when the nucleus was visible and aligned, to ensure that the section plane was appropriate.

### Immunohistochemistry (IHC) analysis

For immunohistochemical evaluations, paraffin sections of zebrafish gastrointestinal tract were rehydrated and neutralized for endogenous peroxidases with 3% hydrogen peroxide for 5 minutes followed by rinsing for 5 minutes in distilled water. For antigen retrieval, slides were incubated in three antigen retrieval solutions: citrate buffer (pH 6.0) for tgfb1a, nfkb, and tlrs; EDTA (pH 8.0) for myd88; and 0.01 M Tris-EDTA buffer (pH 9.0) for anti-cnr1, anticnr2, anti-casp3, and anti-casp1 in a steamer (Black & Decker, Towson, MD) for 20 minutes. Non-specific binding was blocked by incubation of slides for 10 minutes with a proteinblocking agent (Dako, Carpinteria, CA) before application of the primary antibody. All antibodies used were specific for zebrafish, or previously tested and reactive also for this species. Slides were incubated overnight in a moist chamber with the following primary antibodies: polyclonal (pAb) rabbit anti-cnr1 (AB9415, Anti-Cannabinoid Receptor 1 Antibody, Millipore Merck, USA) diluted 1:100; pAb anti-cnr2 (PA5-33445, CNR2/CNR2, Thermo Scientific, Rockford, USA) diluted 1:100; anti-tlr3 (CT) (AS-55356, Z-Fish™, Eurogentec), and anti-tlr9 (IN), (AS-55351, Z-Fish™, Eurogentec), both used at dilution of 1:500; anti-tlr1 (Zebrafish antibodies, Berkeley Heights, USA), and anti-tlr2 (Zebrafish antibodies, Berkeley Heights, USA), both used diluted at 1:50; anti-myd88 (CT) (Z-Fish™), anti-casp3 (p12) (CT) (Z-Fish™), anti-casp1 (Z-Fish™), anti tgfb1a (Z-Fish™), all used at dilution of 1:50; pAb anti-nfkb p65 - N-terminal (ab177895, ABCAM), at dilution of 1:200; and pAb anti-nos2a (610332, BD Pharmingen, San Diego, CA) used at dilution of 1:500.

The immunoreaction with streptavidin–immunoperoxidase (Streptavidin–immunoperoxidase, Black & Decker, Towson, MD) was visualized with 3,3′-diaminobenzidine substrate (Vector, Burlingame, UK). Tissues were counterstained with Mayer’s hematoxylin. For negative immunohistochemical controls the primary antibodies were omitted. Sections of zebrafish and killifish spleen and kidney served as positive control tissues for TUNEL, casp1 and 3, tgfb1a, and myd88 cell staining. Positive control tissues for tlrs staining consisted of fish skin and gills sections. For scoring of intestinal parameters such as cnr1- and cnr2-receptor positive cells, tlrs, tgfb1a+, myd88+, nos2a+, and nfkb+ cells, the cells were quantified in the small intestinal compartment of the GI tract (anterior portion of the intestine: villi, basal crypt area, villuscrypt junction) as previously reported^21^. Similar evaluations were performed in an attempt to evaluate the Caspase3, Caspase1 and TUNEL+ cells. All cell types were evaluated using a light microscope (Carl Zeiss, Jena, Germany), a × 40 objective, a × 10 eyepiece, and a square eyepiece graticule (10 × 10 squares, having a total area of 62,500 μm^2^). Ten appropriate fields were chosen for each compartment and arithmetic means were calculated for each intestinal region. Results were expressed as IHC positive cells per 62,500 μm^2^. For all parameters, cells on the margins of the tissue sections were not considered for evaluation to avoid inflation of positive cell numbers.

For the evaluation of different cnr1 and 2 and tlrs subsets in the same histological sections, consecutive 3-μm-thick bioptic cross-sections were cut. Sections were placed consecutively on each of eight separate slides, after which the ninth section was placed on the first slide, next to the first section, continuing for 48 sections. A single slide, upon which were six bioptic cross-sections from each fish, was analysed for any given immunostain. Numbers of cnr1+, cnr 2+, tlrs (1,2,3,9)+, tgfb1+, casp3+ and cas1+ epithelial and/or immunocompetent cells were quantified by using an image analysis system consisting of a light microscope (Carl Zeiss, Jena, Germany) attached to a Javelin JE3462 high-resolution camera and a personal computer equipped with a Coreco-Oculus OC-TCX frame grabber and high-resolution monitor. Computerized color image analysis was performed using Image-Pro Plus software (Media Cybernetics). The area of each biopsy in all six cross-sections in every fish was recorded, as was the total number of epithelial positive cells determined by immunostaining as previously described. For each fish, the total immunostained cells were counted per section, and stained cell densities were expressed as the number of immunocompetent/epithelial cells per square millimeter of analyzed bioptic area^65^.

### RNA extraction and cDNA synthesis

Total RNA extraction from intestinal tissues was performed following Falcinelli and coworkers^66^. Total RNA extracts were eluted in 30 μl of RNAse-free water. Final RNA concentrations were determined with a NanoDrop^TM^ 1000 Spectrophotometer (Thermo Scientific) and the RNA integrity was verified by ethidium bromide staining of 28 S and 18 S ribosomal RNA bands on 1% agarose gel. RNA was stored at −80 °C until use. Total RNA was treated with DNAse (10 IU at 37 °C for 10 min, MBI Fermentas). A total amount of 1 µg of RNA was used for cDNA synthesis, employing the iScript cDNA Synthesis Kit (Bio-Rad).

### Real time PCR

PCRs were performed with the SYBR green method in an iQ5 iCycler thermal cycler (Bio-Rad Laboratories) following Gioacchini and coworkers^67^. Actin beta (*act1b*) and ribosomal protein, large, P0 (*rplp*) were used as internal standards in each sample in order to standardize the results by eliminating variation in mRNA and cDNA quantity and quality. No amplification products were observed in negative controls and no primer-dimer formations were observed in the control templates. The data obtained were analysed using the iQ5 optical system software version 2.0 (Bio-Rad) including GeneEx Macro iQ5 Conversion and genex Macro iQ5 files. The primer sequences used are reported in Table 2S.

### Statistical analysis

Data are presented as mean ± SD. Student’s t-test was used for comparison between the two experimental groups for *in vivo* experimentation. Two-Way ANOVA followed by the Tukey test as a multiple comparisons test was used for comparison among experimental groups for *ex vivo* experimentation. Statistical evaluation of enterocyte length, villus height, crypt depth and villus height/crypt depth ratio were tested by variance analysis and confirmed by nonparametric methods (Wilcoxon). All statistical analyses were performed using the statistical software package Prism5 (Graphpad Software, Inc. USA) with significance accepted at P < 0.05.

## Electronic supplementary material

Table 1S and Table 2S
