# Supplementary material for: Host-probiotic interaction: new insight into the role of the endocannabinoid system by in vivo and ex vivo approaches
Source: Sci Rep. 2017 Apr 28;7:1261. doi: 10.1038/s41598-017-01322-1 (PMC5430882; doi:10.1038/s41598-017-01322-1)
Supplement: Supplementary file 1 — Table 1S and Table 2S [file 41598_2017_1322_MOESM1_ESM.pdf]

Host-probiotic interaction: new insight into the role of the endocannabinoid system by in vivo and ex vivo approaches.

Giorgia Gioacchini[1], Giacomo Rossi[2t], and Oliana Carnevali[1,3t *]

1.Dipartimento Scienze della Vita e dell'Ambiente, Università Politecnica delle Marche, Via Brecce Bianche, 60131 Ancona, Italy;

2.Scuola di Bioscienze e Medicina Veterinaria, Università degli Studi di Camerino, Via Fidanza 15, 62024 Matelica (MC), Italy.

3.INBB Consorzio Interuniversitario di Biosistemi e Biostrutture, 00136 Roma, Italy.

Histology	Control	VSL#3	P value
**Enterocytes length**	37.82 ± 1.97 µm	48.31 ± 1.93µm	<0.001
**Villi length**	0.839 ± 0.085 µm	1.200 ± 0.146 µm	<0.001
**Crypt depth**	0.088±0.081	0.100±0.022	0.202
**Villi/cryptae ratio**	12.8±3.73	14.2±5.51	0.016

Table 1S: Tabulation and statistical evaluation of enterocyte length, villus height, crypt depth and villus height/crypt depth ratio found in the front intestinal tracts. Values indicate mean ± SD, statistical differences were tested by variance analysis and confirmed by non-parametric methods (Wilcoxon).

**Gene**	**Forward**	**Reverse**
cnr1	TCTGTGGGAAGCCTGTTTC	ACCGAGTTGAGCCGTTTG
cnr2	TCAGAGAGACGATGCTAACC	CAAAAGGCTTCCCACAGAG
trpv1	TGATCGTCGCTGGTGCTT	GACTGGGCTCTCTCTGAACG
faah	CATTCTCGGACCAGCCTTCA	CCTTCACTGCCTGTTGGAAGA
abhd4	GAAGAGCAGTTTGTTTCCTCCATAG	GACTCACTCTTTCTGGGTATTGGAT
mgll	CGAGAGGCCGCAGGATTTTA	TGAGTTTAGGAGCCAAGCG
Il1	GCTGGGGATGTGGACTTC	GTGGATTGGGGTTTGATGTG
tnfa	TTGTGGTGGGGTTTGATG	TTGGGGCATTTTATTTTGTAAG
myd88	CAGCGACATCCAGTTTGTGC	GCGGCACCTCTTTTCGATGA
Il10	GCCGTGGAGCAGGTGAAG	GAAGATGTCAAACTCACTCATGGCT
casp1	AGAAAACGACTGGCCTTGCT	TGAGCAAAGTCCCTGATGGC
nos2a	CAAACTCTCTACAGCGGCGG	GAGTACAGACGGGGCTTCAG
tgfb1a	CGTCTTCCAGCAAGCTCAGA	TGGAGACAAAGCGAGTTCCC
nfkb	GGCCAAAGACACTGTTCGGA	AGGACTTGCGGTTCTTCTCG
tlr1	CGGGAAAAACCATCGTGCAA	TAGTGGCACCATTCGCTCTG
tlr2	CTGTCGGAGCACTTTGTGAG	CCACAACAGCTTAACACTCGTC
tlr3	GACTACGTGATAGCTCCGCC	ACAAGCGTAGAACAAGGGCA
tlr9	GGATTCCTGGAGTCTCGTGC	CTGCTGCACCAAAAGGAAGG
casp3	CCGCTGCCCATCACTA	ATCCTTTCACGACCATCT
baxa	GGCTATTTCAACCAGGGTTCC	TGCGAATCACCAATGCTGT
bcl2a	CCTTCAATAAAGCAGTGGAGGAA	CGGGCTATCAGGCATTCAGA
act1b	GGTACCCATCTCCTGCTCCAA	GAGCGTGGCTACTCCTTCAC
rplp	CTGAACATCTCGCCCTTCTC	TAGCCGATCTGCAGACACAC

Table 2S: Lists of primers used in gene expression analyses by Real-Time qPCR.
